# Evaluation of adnexal masses with ultrasonographic parameters and magnetic resonance imaging

**DOI:** 10.1007/s00404-025-07945-4

**Published:** 2025-07-04

**Authors:** Cem Erdoğan, Ümmihan Topal, Engin Çelik, İsmail Özdemir

**Affiliations:** 1 Department of Obstetrics and Gynecology, Health Sciences University Istanbul Kanuni Sultan Süleyman Training and Research Hospital, Istanbul, Turkey; 2 Department of Radiology, Health Sciences University Istanbul Kanuni Sultan Süleyman Training and Research Hospital, Atakent Mh, Turgut Özal Bulvari No: 46/1, 34303 Küçükçekmece, Istanbul, Turkey

**Keywords:** Ovarian neoplasms, Ultrasonography, Magnetic resonance imaging

## Abstract

**Purpose:**

Adnexal masses (AMs) are commonly seen gynecological problems. Most of the AMs of women in reproductive period are physiologic. A rare but lethal cause of AMs is ovarian cancer. It is important to distinguish benign and malignant AMs. In this study, two scoring systems named “Evaluation of Different Neoplasms in Adnexa (ADNEX)” model and “Ovarian-Adnexal Reporting Data System MR (O-RADS MR)” were examined in terms of diagnostic performance in distinguishing benign or malignant AMs.

**Methods:**

Patients undergone surgery due to AMs were involved in this retrospective study. ADNEX risk model scores and MRI results of patients were re-evaluated for calculating O-RADS MRI scores.

**Results:**

284 patients enrolled in this study. ADNEX risk model had a 93.8% (95% CI: 90.9–96.7%) of area under the ROC curve (AUC) for malignancy risk (*p* < 0.001). O-RADS risk model had a 95.7% (95% CI: 92.8–98.6) of AUC (*p* < 0.001). When cut-off value was set as 42%, sensitivity and specificity of ADNEX risk model were 87% and 88.6, respectively. Sensitivity and specificity of O-RADS MRI risk scoring system were 93.8% and 93.2 when cut-off value was set as ≥ 4, respectively. AUC values of ADNEX risk model and O-RADS MRI scores were not significantly different in terms of differentiating between benign and malignant cases (*p* = 0.218).

**Conclusion:**

ADNEX risk model and O-RADS MRI score are successful in terms of identifying benign and malign cases for evaluation of AMs. There was no significant difference in the ability of these two methods to distinguish benign and malignant cases.

**Supplementary Information:**

The online version contains supplementary material available at 10.1007/s00404-025-07945-4.

## What does this study add to the clinical work

**Table:** 

Our study is important, because it is also a validation study for the O-RADS MRI risk classification system.
There is no difference in AUC values when ADNEX risk model and O-RADS MRI score are compared in terms of their power to differentiate between benign and malignant cases (*p* = 0.218).
In the evaluation of adnexal masses may alleviate the cost burden by reducing the use of MRI. The use of O-RADS MRI not only contributes to the development of a common language between disciplines, but also contributes to the optimized management of lesions when applied in selected cases, such as lesions that cannot be characterized on ultrasound.

## Introduction

Adnexal masses (AM) are one of the very commonly seen gynecological problems. Most of the AMs of women in reproductive period are physiologic [1]. A rare but quite lethal cause of AMs is ovarian cancer; however, 5-year survival rate is over 90% if it is diagnosed at early stages [2, 3].

Anamnesis, physical examination, imaging modalities, and tumor markers are suggestive in evaluation of AMs. Although ultrasonographic examination is commonly used as a first-line imaging method, further imaging methods and scoring systems are needed for differentiating between benign and malignant lesions for approximately one of the four AMs [4, 5]. For this reason, scoring models, such as Risk of Malignancy index 1990 [6], Risk of ovarian malignancy algorithm 2010 [7], The Multivariate index assay 2009 [8], and Simple rules 2008 [9], have been developed. Assessment of Different Neoplasms in the Adnexa (ADNEX) model was developed in 2014 by the International Ovarian Tumor Analysis (IOTA) group. Risk score is calculated using nine parameters according to this risk model group. This risk model provides the chance of being benign or malignant masses, and the probability percentage of being borderline, stage I, stage II–IV, or secondary metastatic ovarian cancer in malignant tumors along with relative risks of these categories [10, 11].

Ovarian-adnexal reporting data system magnetic resonance imaging (O-RADS MRI) is another method for evaluating AMs. MRI is a good choice for diagnosis of AMs identified during ultrasonographic evaluation without a clear differentiation between benign or malignant nature as well as risk stratification. Dynamic, multiphase, and contrast-enhanced MRI successfully identifies the nature of AM due to the contrast agent resolution [12]. O-RADS MRI system was developed by Thomassin-Naggara et al. in 2020. This scoring system is based on MRI features with high positive and high negative predictive values for differentiating between benign and malignant masses. American College of Radiology (ACR) has clearly suggested the definition and the utilization of respective parameters [12–14]. It is scored between 1 and 5 according to these features.

In this study, we evaluated the performances of the ADNEX risk model scoring system, which is easy to use due to the wide accessibility of ultrasonography and laboratory parameters, as well as the O-RADS MRI scoring system, which has recently become increasingly popular. This study is important, because it is a validation study for the O-RADS MR risk classification system. Our aim in this study is to contribute to the optimal management of AMs by comparing the ADNEX risk model and O-RADS MR score in terms of their success in differentiating benign and malignant lesions. As far as we know, no studies have been conducted for this purpose in the literature. Our main goal is not to compare ultrasonography and MRI directly. Our second aim is to contribute to a consensus on a similar language between radiologists and clinicians by preventing unnecessary operations and additional treatments, promoting the treatment of patients with malignant AMs in competent reference centers, and improving the standardization of methods used in evaluation.

## Materials and methods

### Study design and ethical approval

This study was approved by the ethical committee of our hospital and informed consent form for every patient has been renounced (study number, 2020-222). Digital data of 1515 patients undergone surgery for AM in our center between December 2017 and February 2020 were screened for retrospectively investigating pre-operative malignancy risk and comparing histopathological results on data management system of our hospital. A total of 284 patients who were eligible for the study protocol were involved in this study.

Inclusion criteria were patients aged over 18 years who underwent surgery due to AM with pathology report, presence of tumor marker (CA-125) measurement, presence of dynamic contrast-enhanced abdominal MRI, and ultrasonographic evaluation performed in 120 days of pre-operative period. Patients without AMs, patients with known gynecological malignancy before operation, patients whose AMs were planned to be followed up, patients without dynamic contrast-enhanced abdominal MRI and ultrasonographic evaluation, and patients without a measurement of CA-125 levels were excluded from the study.

Age, gravity, parity, menopausal status, symptoms, physical examination findings, personal background, family history, CA-125 levels, and ultrasonographic findings of all patients were recorded. For ultrasonography, patients examined with gray-scale transvaginal and abdominal transducers of GE Voluson 730 Expert (2004, Austria) and Mindray Diagnostic Ultrasound System DC-7 (2012, Germany) ultrasonography devices were included. For MRI, patients examined with 1.5 T morphologic sequences (T2-weighted fat-suppressed/not-suppressed, T1-weighted fat-suppressed/not-suppressed, diffusion-weighted imaging, apparent diffusion coefficient maps, and dynamic T1-weighted subtraction after intravascular gadolinium injection) and functional sequences of Siemens Magnetom Avanto (2014, Germany) device were included.

Patients’ ADNEX risk model scores were calculated retrospectively using ultrasonographic evaluation findings, blinded to histopathological results, through the calculation section on the official IOTA website (www.iotagroup.org/sites/default/files/adnexmodel). In this area, three clinical parameters and six USG parameters were filled. Three clinical parameters were age, presence or absence of clinical evaluation in an oncology clinic, and CA-125 level. Six ultrasonographic evaluation parameters filled for every patients were maximum lesion diameter, maximum solid lesion diameter, presence or absence of more than ten locules, number of papillary projections (absent, one, two–three, more than three), and presence or absence of acoustic shadowing and ascites. Probability of being benign, malignant, and borderline were obtained as well as probability of being stage 1, stage 2–4, and metastatic cancer for patients with malignancy (Figure S1).

Patients’ abdominal MRI images were evaluated retrospectively by a radiologist with 14 years of experience in abdominal imaging, who calculated the O-RADS MR scores without knowledge of the histopathological results. O-RADS MRI risk classification system published by the ACR was used for calculating O-RADS MRI scores [14].

### Standard reference

Origin of masses and status of being benign/malignant/borderline were classified by evaluating histopathology results. Borderline tumors were included in the malignancy category. Stages of malignant tumors were identified by the FIGO criteria updated at the January 1, 2014 [15]. Histopathological diagnosis was considered as the golden standard for evaluating the data. Performances of ADNEX risk model and O-RADS MRI scores were also evaluated [10–12].

### Statistical analysis

All of statistical calculations were performed using R software (version 4.0.5) (R: A language and environment for statistical computing. R Foundation for Statistical Computing, Vienna, Austria, Available online: http/www.r-project.org/). Receiver-Operating Characteristic curve (ROC) analysis, calculation of area under the curve (AUC), identification and comparison of the confidence intervals, calculations of the sensitivity/specificity, and appropriate cut-off points were performed with the pROC package [16]. Optimal cut-off points were identified according to the Youden index. Compliance of the variables with the normal distribution were evaluated with the Kolmogorov–Smirnov and Shapiro–Wilk test along with *Q*–*Q* plot and histogram graphics. Continuous data without normal distribution were presented as median (interquartile range). Categorical data were presented with frequency (percentage). Due to lack of normal distribution in continuous data, they were analyzed with the Mann–Whitney *U* test. Categorical data were compared with Pearson’s Chi-square test if the number of observation was adequate, whereas they were compared with Fisher’s exact test if the number of observation was inadequate. Due to inadequate number of observation, Pearson Chi-square test was applied after eye merge in the cases with more than two categories. *p* values lower than 0.05 were considered significant.

## Results

### Demographics

Age range of 284 enrolled patients was between 18 and 88 years with a median value of 44 (34–53). Median gravida value was 3 (1–4) and median parity value was 2 (1–3). Most common symptom was groin/abdominal pain in 172 (60.6%) patients, whereas 44 (15.5%) patients were asymptomatic. In terms of histopathological diagnosis, masses of 219 (77.1%) patients were benign, whereas masses of 65 (22.2%) patients were malignant (Table 1).

**Table 1:** Demographics, histopathologic results, and staging of the patients

Characteristic	
Age	44 (34–53)
Gravida	3 (1–4)
Parity	2 (1–3)
Menopause status *n* (%)	
Premenopause	182 (64.1)
Menopause	102 (35.9)
Clinical presentation *n* (%)	
None	44 (15.5)
Groin/abdominal pain	172 (60.6)
Abnormal uterine bleeding	24 (8.5)
Abdominal swelling/palpable mass	40 (14.1)
Other	4 (1.4)
Histopathology results *n* (%)	
Benign	219 (77.1)
Malign	65 (22.9)
Histopathological subtype *n* (%)	
Physiological and non-neoplastic masses	7 (2.5)
Endometrioma	42 (14.8)
Epithelial benign tumors (serous, mucinous, seromucinous cystadenoma and cystadenofibroma)	108 (38)
Epithelial borderline tumors (serous, mucinous and seromucinous type)	10 (3.5)
Epithelial malignant tumors (serous, mucinous, seromucinous, clear cell, endometrioid, mixed type)	42 (14.8)
Germ cell tumor (mature cystic teratoma)	28 (9.9)
Benign sex cord-stromal tumor (leiomyoma, fibrothecoma, sclerosing stromal tumor)	20 (7)
Malignant sex cord-stromal tumor (granulosa cell tumor, sertoli-ledding cell tumor, sarcoma)	11 (3.9)
Infectious masses (TOA, salpingitis, hydrosalpenx)	12 (4.2)
Metastatic masses (endometrium and colon cancer)	2 (0.7)
Other benign audiences (hemangioma, schwannoma)	2 (0.7)
Staging *n* (%)	
Benign	219 (77.1)
Borderline	10 (3.5)
Stage 1	26 (9.2)
Stage 2–4	27 (9.5)
Metastatic	2 (0.7)

Median age of patients with benign lesions was 42 (33–50) and median age of patients with malignant lesions was 51 (43–62). There was statistically significant difference between these two groups (*p* < 0.001). 154 (70.3%) and 65 (29.7%) of benign patients were in pre-menopausal and menopausal periods, respectively. 28 (43.1%) and 37 (56.9%) of malignancy patients were in pre-menopausal and menopausal periods, respectively. This difference between malignant and benign groups was statistically significant (*p* < 0.001). In terms of CA-125 values, median values of patients with benign and malignant masses were 20 (12–44.5) U/mL and 127 (29–312) U/mL, respectively. There was a significant difference between these values (*p* < 0.001) (Table 2).

**Table 2:** Comparison of patients who were histopathologically diagnosed with benign and malignant lesions in terms of age, menopausal status, CA-125, and ultrasonographic features

Characteristic	Benign (219)	Malign (65)	*p* value
Age^a^	42 (33–50)	51 (43–62)	< 0.001^b^
Menopause status *n* (%)			0.001^c^
Premenopause	154 (70.3)	28 (43.1)	
Menopause	65 (29.7)	37 (56.9)	
CA 125 (U/mL)^a^	20 (12–44.5)	127 (29–312)	< 0.001^b^
*US features*			
Lesion diameter (mm)^a^	80 (63–120)	100 (80–150)	0.004^b^
Solid structure diameter (mm)^a^	0 (0–20)	45 (28–73)	< 0.001^b^
More than 10 locules *n* (%)			0.346^d^
Exist	10 (4.6)	5 (7.7)	
None	209 (95.4)	60 (92.3)	
Number of papillary structures *n* (%)			< 0.001^c,e^
None	168 (76.7)	29 (44.6)	
1	34 (15.5)	3 (4.6)	
2	14 (6.4)	9 (13.8)	
3	1 (0.5)	9 (13.8)	
More than 3	2 (0.9)	15 (23.1)	
Acoustic shadowing *n* (%)			0.026^d^
Exist	15 (6.8)		
None	204 (93.2)	65 (100)	
Ascites *n* (%)			< 0.001^d^
Exist	2 (0.9)	8 (12.3)	
None	217 (99.1)	57 (87.7)	

^a^Median, U/mL: units per milliliter, mm: millimeter

^b^Mann–Whitney *U* test

^c^Pearson chi-squared test

^d^Fisher’s definitive test

^e^For some data, grouping as “None-Exist” was performed due to insufficient number of observations

### Ultrasonography findings

In terms of ultrasonographic imaging features, median values for lesion diameters were 80 (63–120) and 100 (80–150) for benign and malign lesions, respectively. Difference between these two groups was statistically significant (*p* = 0.004). Median values of solid mass diameters of benign and malignant cases were 0 (0–20) mm and 45 (28–73) mm, respectively. Difference between these two groups was significant (*p* < 0.001) (Table 2).

In benign cases, more than ten locules were present in ten (4.6%) cases, whereas less than ten locules were present in 209 (95.4%) cases. In malignant cases, more than 10 locules were present in 5 (7.7%) cases whereas less than 10 locules were present in 60 (92.3%) cases. There was no significant difference between benign and malignant cases in terms of number of locules (*p* = 0.346) (Table 2).

There was no papillary structure in 168 (76.7%) benign cases and 29 (44.6%) malignant cases. There was significant difference between these two groups in terms of presence of papillary structure (*p* < 0.001) (Table 2).

Acoustical shadowing was present in 15 (6.8%) benign cases, whereas it was not present in any malignant cases. There was significant difference between these two groups in terms of acoustical shadowing (*p* = 0.026) (Table 2).

Ascites was present in 2 (0.9%) benign cases, and 8 (12.3%) malignant cases. Difference between two groups was significant (Table 2).

### ROC curves and analysis

**Fig. 1:**
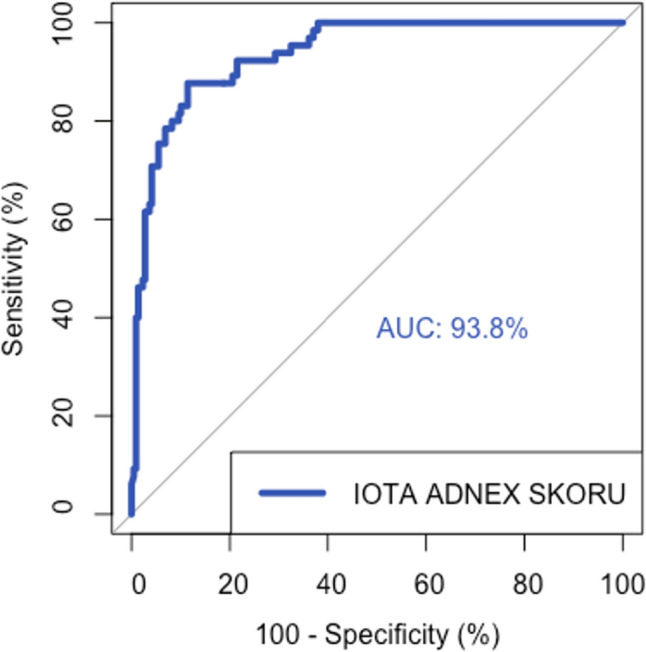
ROC curve of total malignancy risk in ADNEX risk model

In terms of total malignancy risk, AUC for ADNEX risk model was 93.8% (95% CI: 90.9–96.7%) and it has good differentiation between benign and malignant cases (*p* < 0.001) (Fig. 1).

**Fig. 2:**
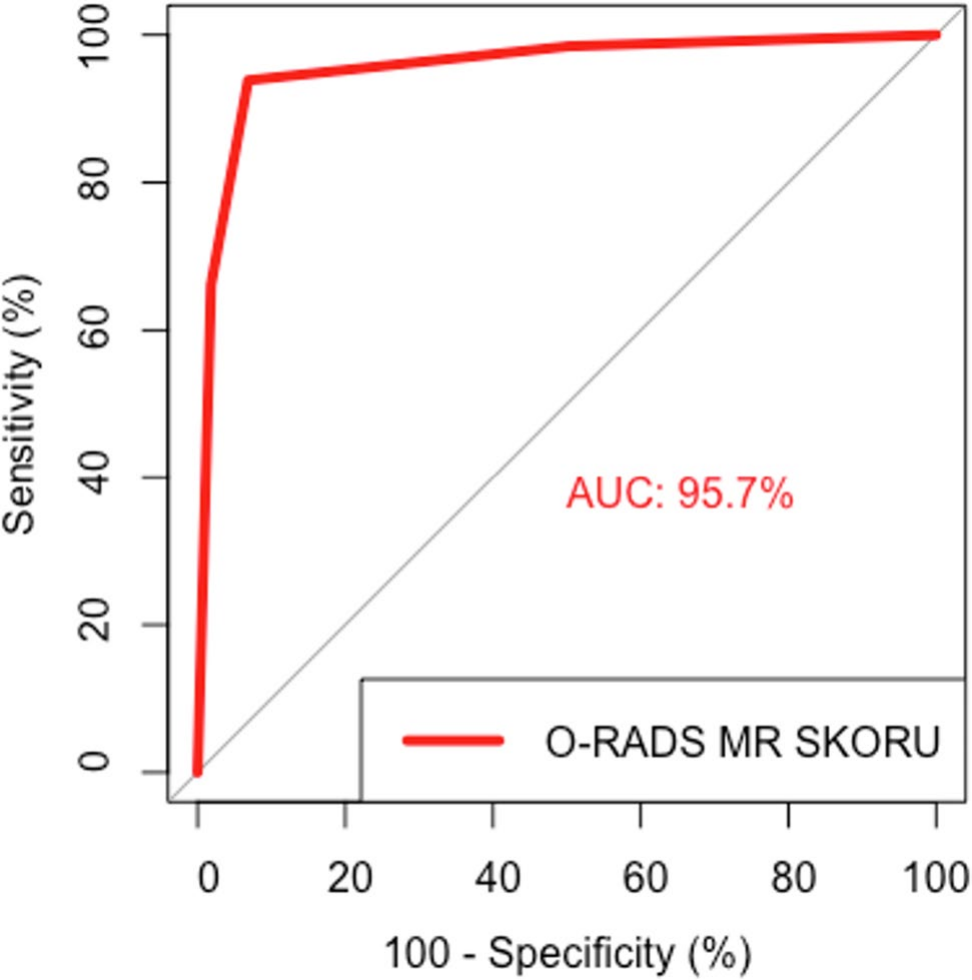
ROC curve of total malignancy risk in O-RADS MRI score

In terms of total malignancy risk, AUC for O-RADS MRI score was 95.7% (95% CI: 92.8–98.6%), and it has good differentiation between benign and malignant cases (*p* < 0.001) (Fig. 2).

**Fig. 3:**
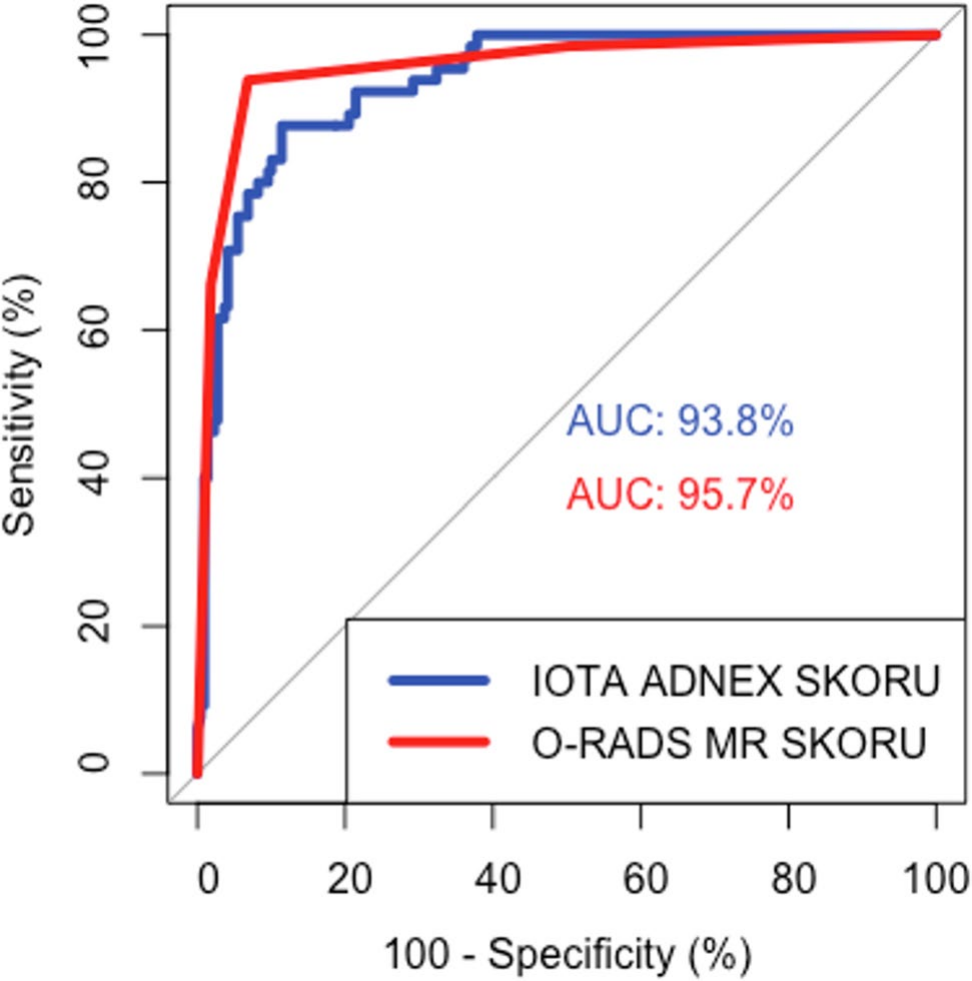
Comparison of ROC curves of total malignancy risk in ADNEX risk model and O-RADS MRI score

In terms of differentiation power between benign, and malignant cases, there was no difference between AUC values of ADNEX risk model and O-RADS MRI score (*p* = 0.218) (Fig. 3).

Sensitivity and specificity values were calculated in ROC analysis of ADNEX risk model for all malignancy cut-off points. Then sensitivity, specificity, positive predictive value (PPV), negative predictive value (NPV), positive likelihood ratio [LR(+)], negative likelihood ratio [LR(−)], and validity coefficient values of determined 10%, 25%, 42%, 50%, and 99% points were calculated. According to the results, point of 42% malignancy risk value was the optimal cut-off point. Point of 10% had the highest sensitivity and the point of 99% had the highest specificity, whereas point of 25% had a relatively high sensitivity and point of 50% had a relatively high specificity (Table 3).

**Table 3:** Diagnostic performance of ADNEX risk model at the presented cut-off points of malignancy risk

Cut off	Sensitivity	Specificity	PPV	NPV	LR(+)	LR(−)	Accuracy
%10	98.5	63	44.1	99.3	2.66	0.02	71.1
%25	92.3	77.6	55.1	97.1	4.13	0.1	81
%42	87.7	88.6	69.5	96	7.68	0.14	88.4
%50	80	90.9	72.2	93.9	8.76	0.22	88.4
%99	7.7	99.5	83.3	78.4	16.85	0.93	78.5

*PPV* positive predictive value, *NPV* negative predictive value, *LR(*+*)* positive likelihood ratio, *LR(−)* negative likelihood ratio

To demonstrate the diagnostic performance of O-RADS MRI at different cut-off points, three cut-off points were identified in the ROC analysis. At these points, sensitivity, specificity, positive predictive value (PPV), negative predictive value (NPV), positive likelihood ratio [LR(+)], negative likelihood ratio [LR(−)], and validity coefficient values were calculated (Table 4). Score of ≥4 was identified as the optimal cut-off point, whereas score of 3 had high sensitivity and score of 5 had high specificity.

**Table 4:** Progressive cut-off for overall risk of malignancy using the O-RADS MRI categories in 284 patients

	Sensitivity	Specificity	PPV	NPV	LR(+)	LR(−)	Accuracy
ORADS ≥ 3	98.5	49.8	36.8	99.1	1.96	0.03	60.9
ORADS ≥ 4	93.8	93.2	80.3	98.1	13.70	0.07	93.3
ORADS = 5	66.2	98.2	91.5	90.7	36.22	0.34	90.8

*PPV* positive predictive value, *NPV* negative predictive value, *LR(*+*)* positive likelihood ratio, *LR(−)* negative likelihood ratio

The number of false-positive and false-negative cases and their characteristics in grouped cases were identified according to the determined optimal cut-off points (42% for the ADNEX risk model, ≥4 for the O-RADS MRI score).

Twenty five (75.8%) and eight (24.2%) of misdiagnoses at the optimal cut-off point of ADNEX risk model were false positive and false negative, respectively. Fifteen (78.9%) and four (21.1%) of misdiagnoses at the optimal cut-off point of ADNEX risk model were false positive and false negative, respectively.

In the histopathologic diagnoses of patients classified as false positives, 1 (4%) of the false-positive cases in the ADNEX risk model were endometriomas, 7 (28%) were epithelial benign tumors, 3 (12%) were mature cystic teratomas, 13 (52%) were benign sex cord-stromal tumors, and 1 (4%) were other benign masses. In the O-RADS MRI group, 5 (33.3%) of the false-positive cases were epithelial benign tumors, 1 (6.7%) was a mature cystic teratomas, 6 (40%) were benign sex cord-stromal tumors, 1 (6.7%) was an infectious mass, and 2 (13.3%) were other benign masses. In ADNEX risk model group, histopathologic classification of false-negative cases were as follows: 2 (25%) epithelial borderline tumor, 1 (12.5%) epithelial malignant tumor, 4 (50%) malignant sex cord-stromal tumor, and 1 (12.5%) metastatic mass. However, four of all false-negative cases were all epithelial borderline tumors in O-RADS MRI group.

## Discussion

In this study, when the cut-off point for the ADNEX risk model was set at 10%, the sensitivity was found to be 98.5% and the specificity was 63%. This point was the cut-off point with the highest sensitivity. At the cut-off point of 42%, the sensitivity was found to be 87.7% and the specificity was 88.6%. This point was the optimal cut-off point for highest sensitivity and specificity. At the cut-off point of 99%, the sensitivity was found to be 7.7% and the specificity was 99.5%. This point was the point with highest specificity. AUC of total malignancy risk in ADNEX risk model was 93.8% (95% CI: 90.9–96.7%) and it has good differentiation power between malignant and benign cases (*p* < 0.001). Sensitivity and specificity of O-RADS MRI data at the cut-off value of ≥4 were 93.8% and 93.2%, respectively. This point was considered as the optimal cut-off value. AUC of O-RADS MRI score was 95.7% (95% CI: 92.8–98.6%) and it has good differentiation power between malignant and benign cases (*p* < 0.001).

In the literature, study of Van Calster et al. with the data of 5909 enrolled patients in the year 2014 which the ADNEX risk model was defined at showed that sensitivity was 96.5% and specificity was 71.3% at the cut-off point of 10% for malignancy [2]. In the multi-centered cohort study of Van Calster et al. (2020) with 4905 enrolled patients; ADNEX risk model, risk of malignancy index, simple rules, Logistic regression model 2 were compared with each other. In that study, sensitivity was 91%, and specificity was 85% when the cut-off point was set to 10% for ADNEX risk model, respectively. In that study, ADNEX risk model and simple rules had the best performance [11]. In the multi-centered cohort study of Sayasneh et al., sensitivity was 97% and specificity was 68% when the cut-off point was set to 10%, whereas sensitivity was 86% and specificity was 84% when the cut-off point was set to 30% (18). Studies indicate that ADNEX risk model is a successful method for differentiating between benign and malign nature of AMs. The sensitivity and specificity values obtained at the determined cut-off points in our study were consistent with those of other studies conducted [2, 11, 17].

There are limited number of studies performed for O-RADS MRI risk classification system in the literature. Sensitivity was 93% and specificity was 91% in the article of Thomassin-Naggara et al. in 2020. In our study, sensitivity was 93.8% and specificity was 93.2% for O-RADS MRI when the optimal cut-off point was set to ≥4. Data in our study were in accordance with that study [12].

The management of AMs is not solely dependent on imaging methods; it may also vary according to the patients’ existing risk factors and symptoms related to ovarian cancer, the healthcare policies of the country, and the clinician’s work environment in the hospital. Therefore, we identified different cut-off points for both of two models. It was found that optimal cut-off points were malignancy risk of 42% in ADNEX risk model and score of 4 in O-RADS MRI. Patients at the above this cut-off point have significantly higher risk for malignancy and they all must be evaluated by a specialist of gynecologic oncology. Different cut-off values may be utilized in the settings requiring higher sensitivity or higher specificity according to the clinician’s choice. As an example for considering risk factors of the patients, a point with higher sensitivity may be chosen for a patient with a family history of ovarian cancer. As an example for considering hospital features, a clinician working at a hospital with MRI, available gynecologic oncologist, and frozen pathology examination may prefer higher specificity whereas a clinician without these opportunities may need higher sensitivity.

The AUC values of the ADNEX risk model and O-RADS MRI score were not significantly different in differentiating between benign and malignant cases (*p* = 0.218). Therefore, the use of O-RADS MRI should be reserved for selected cases. When the O-RADS MRI scores of the 25 patients in the false-positive group were evaluated at the cut-off point of 42% for the ADNEX risk model, 20 (80%) had scores of 2 or 3, while 5 (20%) had scores of 4 or 5. When the O-RADS MRI scores of the 8 false-negative patients in the ADNEX risk model were evaluated, 1 (12.5%) patient had an O-RADS score of 2, while the other 7 patients had O-RADS scores of 4 or 5.

When evaluating the false-positive and false-negative patients according to the ADNEX risk model score, 13 (52%) of 25 false-positive patients were patients with fibrothechoma and leiomyoma in the benign sex cord-stromal tumor. Four (50%) of eight false-negative patients were in the malign sex cord-stromal tumor group. According to the O-RADS MRI risk classification system, 6 (40%) of 15 false-positive patients were in benign sex cord-stromal tumor group and all of 4 false-negative patients were in borderline epithelial ovarian tumor group.

As a limitation, all of our enrolled patients were among the patients with uncertain differentiation between benign and malignant nature and patients with high predicted malignancy risk who all admitted to our center due to AMs. Due to retrospective nature of our study, we were unable to differentiate these two groups from each other. For similar reasons, patients followed up conservatively and patients with O-RADS MRI score of 1 were not present in our study. The number of patients with metastatic malignancy was very few, because primary treatment of malignancy metastasized to ovaries is not surgery. Retrospectively evaluated ultrasonographic evaluation reports by different clinicians, lack of specified evaluation for ADNEX model, and evaluation of all MRI images by the same clinician are other limitations of our study. To our knowledge, there has been no reported clinical study comparing ADNEX risk model score and O-RADS MRI score. Therefore, our findings are important for contributing to the literature.

In conclusion, ADNEX risk model score and O-RADS MRI score are both successful methods for differentiating benign and malignant cases in the AMs. There is no significant difference between these two methods in terms of differentiation power between benign and malignant cases. Utilization of ADNEX risk model may reduce economic burden by decreasing MRI utilization. O-RADS MRI scoring system may benefit selected cases due to its higher sensitivity and specificity. Prospective studies which only includes the patients whose differentiation between benign/malign nature could not be done by ultrasonographic evaluation will optimally demonstrate the performance of scoring systems.

## Supplementary Information

Below is the link to the electronic supplementary material.

**Fig. S1:** An example of ADNEX risk model showing malignancy risk of an adnexal mass based on clinical and ultrasonographic findings (www.iotagroup.org/sites/default/files/adnexmodel). (JPG 186 KB)
