# Supplementary figures and images for: Evaluation of adnexal masses with ultrasonographic parameters and magnetic resonance imaging
Source: Arch Gynecol Obstet. 2025 Jul 4;312(5):1529–37. doi: 10.1007/s00404-025-07945-4 (PMC12589298; doi:10.1007/s00404-025-07945-4)

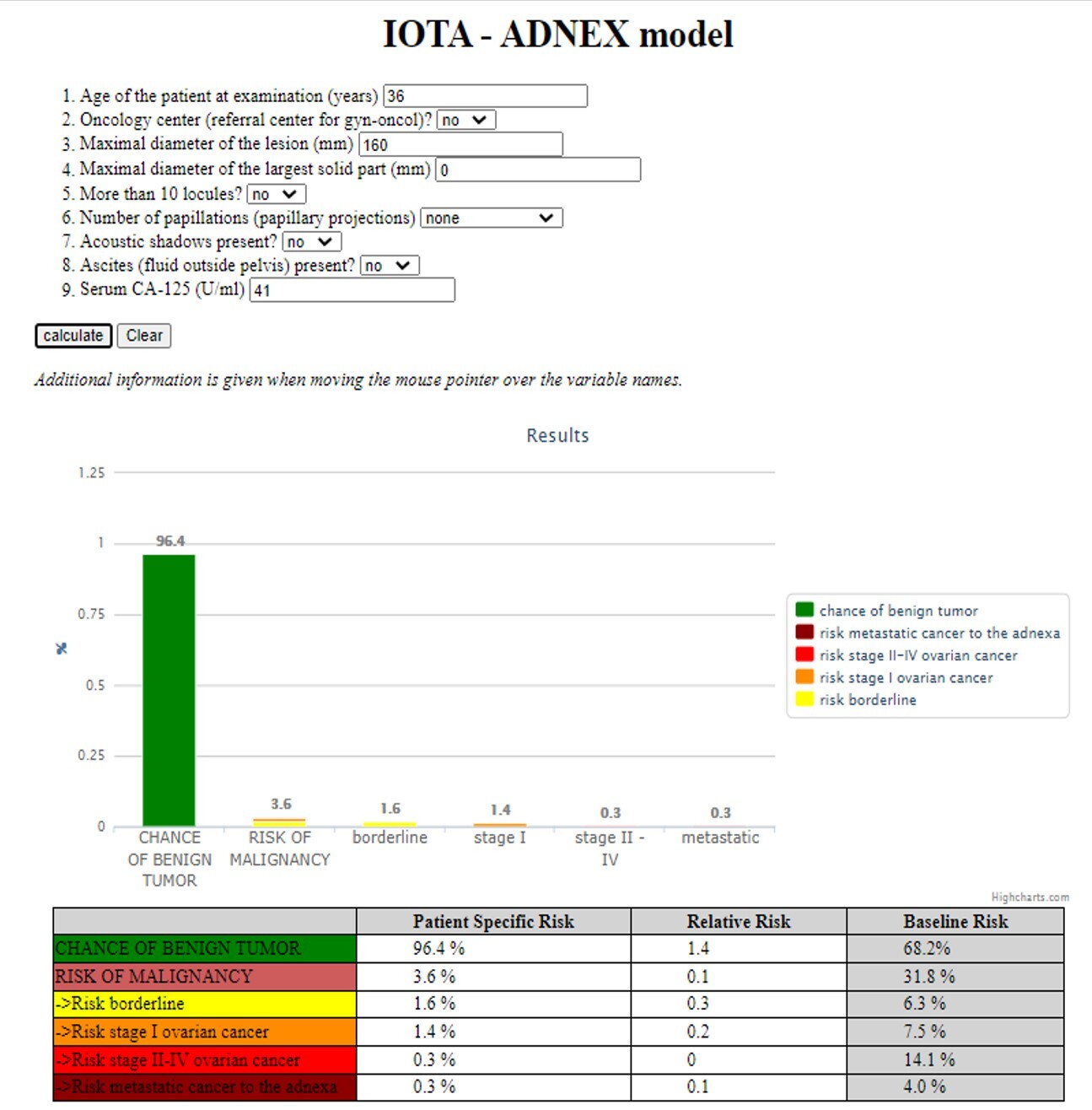

Supplement: Supplementary file 1 — Fig. S1: An example of ADNEX risk model showing malignancy risk of an adnexal mass based on clinical and ultrasonographic findings (www.iotagroup.org/sites/default/files/adnexmodel). (JPG 186 KB) [file 404_2025_7945_MOESM1_ESM.jpg]
